# Genetic trend of the junctional epidermolysis bullosa in the German shorthaired pointer in Italy

**DOI:** 10.1002/vro2.15

**Published:** 2021-08-19

**Authors:** Stefano Frattini, Michele Polli, Matteo Cortellari, Alessio Negro, Arianna Bionda, Jacopo Riva, Rita Rizzi, Stefano Marelli, Paola Crepaldi

**Affiliations:** ^1^ Department of Agricultural and Environmental Sciences‐Production Landscape, Agroenergy University of Milan Milan Italy; ^2^ Department of Veterinary Medicine University of Milan Milan Italy; ^3^ Vetogene srl Milan Italy

**Keywords:** disease control, dog, JEB, molecular genetics

## Abstract

**Background:**

Epidermolysis bullosa (EB) is a hereditary heterogeneous group of mechanobullous disorders caused by mutations in several structural skin proteins observed in both humans and animals. In this work, we report the incidence and the genetic trend of the junctional epidermolysis bullosa (JEB), a major type of EB, in the Italian German Shorthaired Pointer (GSPs) population in a 10 years span.

**Methods:**

In this study, we monitored the genetic trend of JEB in the Italian population of the GSPs from 2009 to 2018 in 750 animals. The studied mutation was the insertion (4818+207 ins 6.5 kb) of repetitive satellite DNA within intron 35 of the *LAMA3* gene.

**Results:**

Allele frequencies showed a reduction of the mutated (C) allele during the years, with the only exception of 2017, when 13 dogs were diagnosed as carrier for the genetic pathology. A regression logistic analysis was performed, including sex, coat colour and their interaction. Our results showed that there was a statistically significant association with coat colour.

**Conclusions:**

The simplicity and the low cost of the analysis for the detection of this pathology suggests that a deeper identification of carrier dogs will allow better breeding strategies and management, leading to a rapid JEB eradication.

## INTRODUCTION

Epidermolysis bullosa (EB) is a hereditary heterogeneous group of mechanobullous disorders caused by mutations in several structural skin proteins observed in both humans and animals.^1^ EB is characterized by dermoepidermal separation, producing various degrees of skin blistering and shearing that impair the life of EB‐affected individuals.^2^ Four major types of EB are currently known, according to their different level of blisters and the location of the aberrant proteins involved: EB simplex (EBS) where detachment occurs in the basal layer of the epidermis; dystrophic EB (DEB) in which defects are due to a deficit of type VII collagen, a major component in the skin basement membrane; Kindler syndrome, characterized by poikiloderma, trauma‐induced skin blistering, mucosal inflammation, and photosensitivity; and junctional EB (JEB) in the lamina lucida of the dermoepidermal basement membrane.^3^

JEB, specifically, encompasses all of the subtypes of EB that have mechanical fragility ansd blistering in which tissue separation occurs within the lamina lucida of the basement membrane zone at the dermal–epidermal junction. The defective adhesion of the epithelium results from mutations affecting either the extracellular matrix protein laminin 5, its cellular receptor integrin a6b4, or collagen type XVII, a transmembrane component of the hemidesmosomes.^4^ The hemidesmosomes are multi‐protein complexes that play a pivotal role in the stable association of basal epithelial cells to the underlying basement membrane in stratified and pseudostratified epithelia. Mutations in the genes encoding hemidesmosomal proteins and functionally associated structures have been shown to result in different subtypes of JEB. The classic junctional forms are associated with mutations in the laminin 5 genes, LAMA3, LAMB3, and LAMC2; in particular, the Herlitz (lethal) JEB variant frequently displays premature termination codon mutations in these genes, driving severe defects in epithelial cell adhesion and death soon after birth. The non‐Herlitz (non‐lethal) variant carries mutations in the same genes, but with milder consequences at the mRNA and protein levels, such as missense or splice junction mutations.^5^ JEB is found several mammalian species, such as dog, cat, horse, cattle and sheep.^6^, ^7^, ^8^, ^9^, ^10^, ^11^ The JEB in a specific dog breed, the German Shorthaired Pointers (GSPs), also known as Kurzaar, shows the hallmarks of the condition observed in humans. In GSPs, JEB is caused by an insertion (4818+207 ins 6.5 kb) of repetitive satellite DNA within intron 35 of the *LAMA3* gene. The insertion causes an abnormal mRNA transcript with an insertion of 227 nucleotides in position 4818 at the junction between exon 35 and 36 of the *LAMA3* gene. This 227 bp sequence carries a non‐sense (TAA) codon 33 bp downstream of the insertion site. Considering that conventional treatments are ineffective for homozygous dogs and that heterozygous animals are symptomless, the genetic identification of carrier animals is important.^7^

In humans, EBs are estimated to affect one in 17,000 live births, with 500,000 cases estimated worldwide,^12^ while in several animal species the frequencies of these diseases are still unknown.^13^ Prevalence of inherited JEB in GSPs in Italy was briefly shown in a paper in 2010, however no genetic trends were identified or genotype–phenotype association was found.^14^

In this work, we reported the incidence and the genetic trend of the pathology in the Italian population in a 10 years span. Furthermore, mechanisms of pigmentation are extremely complex, and many cofactors and interactions are still unknown.^15^ However, chromosomal rearrangements could alter molecular interaction, giving unexpected molecular and phenotypic findings. Therefore, we explored the new possibility of phenotype–genotype associations, investigating potential links among coat colours/patterns and the genetic mutation causing the pathology in GSPs.

## MATERIALS AND METHODS

In this study, we monitored the genetic trend of JEB in the Italian population of the GSPs in a period of 10 years (2009–2018).

Samples processing and analysis were carried out by Vetogene SRL, Milano (Italy), one of the official reference labs for the Italian Kennel Club ENCI (FCI associate), who collected all the samples through the years.

All samples were sent for the genetic test analysis by veterinarians who certified the identification of the sample through microchip control. All the owners gave an informed consent for research purpose to the Vetogene laboratory.

The average growth rate on the entire population of GSPs in Italy has been calculated according to the formula:

[(Present/Past)^1/^ *^n^*] − 1

where “Present” is the number of registrations of the last recorded year, “Past” is the number of registrations of the first years and “*n*” is the number of years among the two registrations.

The experimental sample was composed of 750 animals, in which 398 were male and 352 were female. We also collected information about coat colours, considering as phenotypic pattern “solid” when the animals were totally black or brown and “roan” when showing the peculiar mixture of white and pigmented hairs. Coat colour patterns were assigned according to official self‐declarations of breeders storing the samples of the dogs in the Vetogene laboratory. Among male dogs, 217 were considered “roan” and 180 “solid”; among female dogs, 233 were classified as “roan” and 112 as “solid”. For eight tested dogs, it was not possible to collect any coat colour/pattern information.

Since 2008, in collaboration with the Italian Kurzaar Kennel Club (KCI), Vetogene has been able to perform a diagnostic test for JEB in this breed within the country.

Genomic DNA has been extracted either from peripheral blood (Illustra blood genomicPrep Mini Spin Kit; GE Healthcare) or FTA cards (Vet Kard System; Prion Diagnostica), according to the manufacturer's instructions. An allele‐specific PCR method for molecular detection of the causative mutation in the *LAMA3* gene, developed by Polli and others,^16^ has been performed by the laboratory.

### Statistical analysis

To evaluate if there was any difference among the genotypes observed in the first 5‐year period (from 2009 to 2013) and in the second 5‐year period (from 2014 to 2019), we applied a chi‐square test with Yates's correction for continuity.

The prevalence of the genetic pathology was estimated as a percentage by dividing the number of wild‐type and carrier dogs by the total number of dogs analysed.

Here is the classification of the alleles in the *LAMA3* gene: N: null (wild type, no insertion detected); C: carrier (mutated, 6.5 kb insertion detected).

A regression logistic model has been created to analyse the associations between genotype and coat colour:

$$y_{\mathrm{ijk}}\left(\mathrm{genotype}\right)=Sex_{i}+{\mathrm{Coat}}_{j}+e_{ijk}$$

where y_ijk_ = genotype at the (NN or NC), Sex_i_ = male or female; Coat_j_ = the colour of the coat: “solid” (black or brown) or “roan”; and e_ijk_ = random residual effect.

Statistical analysis was performed using the software JMP of SAS (JMP 13.0.0, 2016; SAS Institute Inc., Cary, NC).^17^

## RESULTS

The German Shorthaired Pointer is a medium to large sized breed of dog developed in the 19th century in Germany for hunting. In Italy, this breed is well known and widely reared. Table 1 shows the number of new puppies registered for the breed from 2009 to 2018 in the country.

**TABLE 1:** Population size in Italy (new registration per year)

**Year**	**2009**	**2010**	**2011**	**2012**	**2013**	**2014**	**2015**	**2016**	**2017**	**2018**
**No. of registrations**	3024	3045	2833	2700	2293	2648	2557	2694	2442	2210

Source: https://www.enci.it/libro‐genealogico/razze.

**FIGURE 1:**
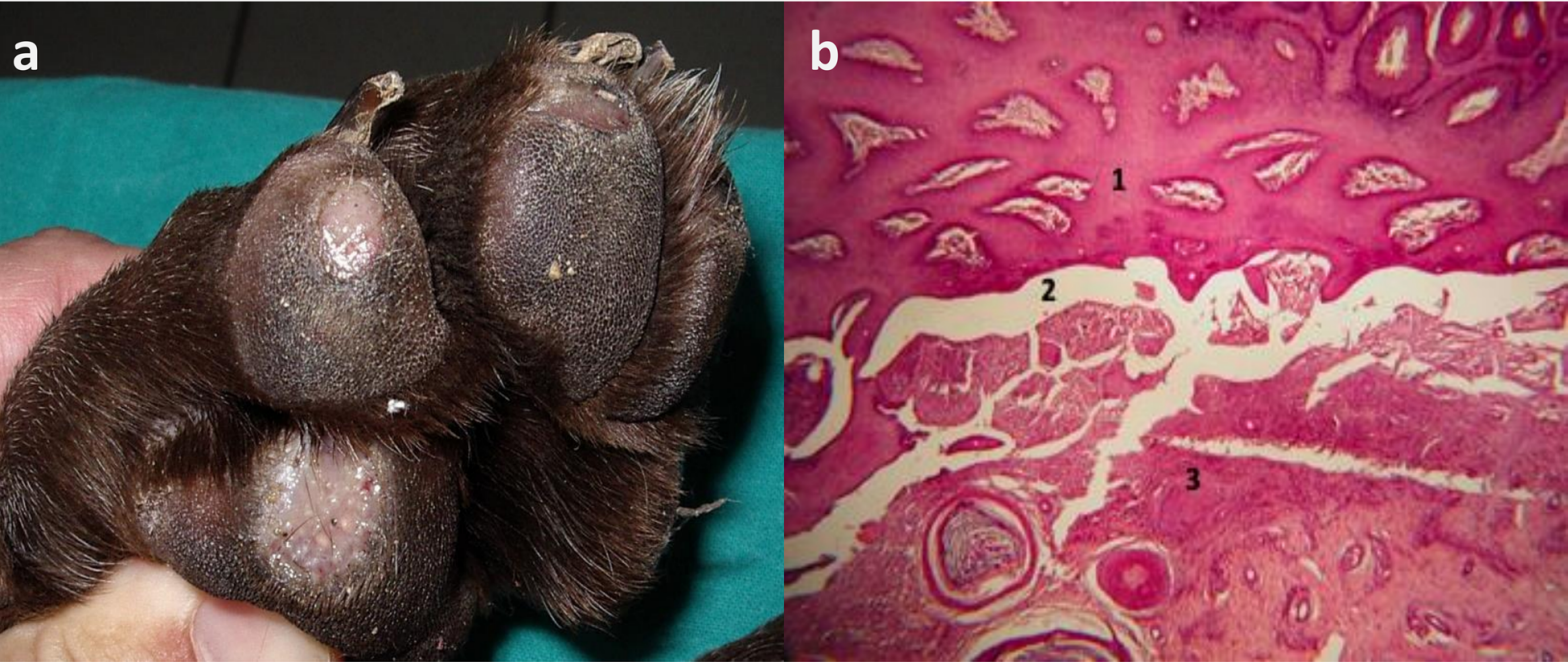
(a) Initial ulcerative erosion of the plantar pad of an affected GSP; (b) plantar pad of a Kurzhaar. A histopathological examination was conducted on the lesions which highlighted the dermoepidermal detachment. 1: epidermis, 2: dermoepidermal detachment, 3: dermis. Hematoxylin–eosin staining (100×)

Inherited JEB has been reported in this breed, exhibiting generalized skin blistering. For an informative and representative scope, Figure 1a and b shows the severity of footpad lesions in an affected subject, from a clinical and a histopathological point of view, respectively.

The genetic laboratory Vetogene performed the tests, highlighting the *LAMA3* gene insertion with an allele‐specific PCR, for 750 GSPs from 2009 to 2018. The number of genetic tests per year, after reaching a maximum peak in 2014 (107 provided tests), decreased in the next 4 years with a minimum of 41 tests provided in 2018 (Table 2).

**TABLE 2:** Number of animals tested, genotypes and allele frequencies at LAMA3 gene locus by year. In the row total, allele frequencies is an average of the 10 years

		**Genotypes**		**Allele frequencies**	
**Year**	**Tested animals**	**NN**	**NC**	**N**	**C**
2009	48	38	10	0.90	0.10
2010	80	66	14	0.91	0.09
2011	84	69	15	0.91	0.09
2012	81	70	11	0.93	0.07
2013	88	73	15	0.91	0.09
2014	107	100	7	0.97	0.03
2015	67	63	4	0.97	0.03
2016	73	66	7	0.95	0.05
2017	81	68	13	0.92	0.08
2018	41	40	1	0.99	0.01
Total	750	653	97	0.94	0.06

Table 2 displays also the genotype and the allele frequency of the tested animals. The NC genotype, for dogs carrying one wild‐type (N) and one mutated (C) allele, showed the highest number^15^ in 2011 and 2013, with the same percentage of the C allele.

Furthermore, allele frequencies showed a reduction of the C allele during the years, with the only exception of 2017, when 13 dogs have been diagnosed as carrier for the genetic pathology. In 2018, the frequency of the C allele reached the minimum value of 0.01.

**FIGURE 2:**
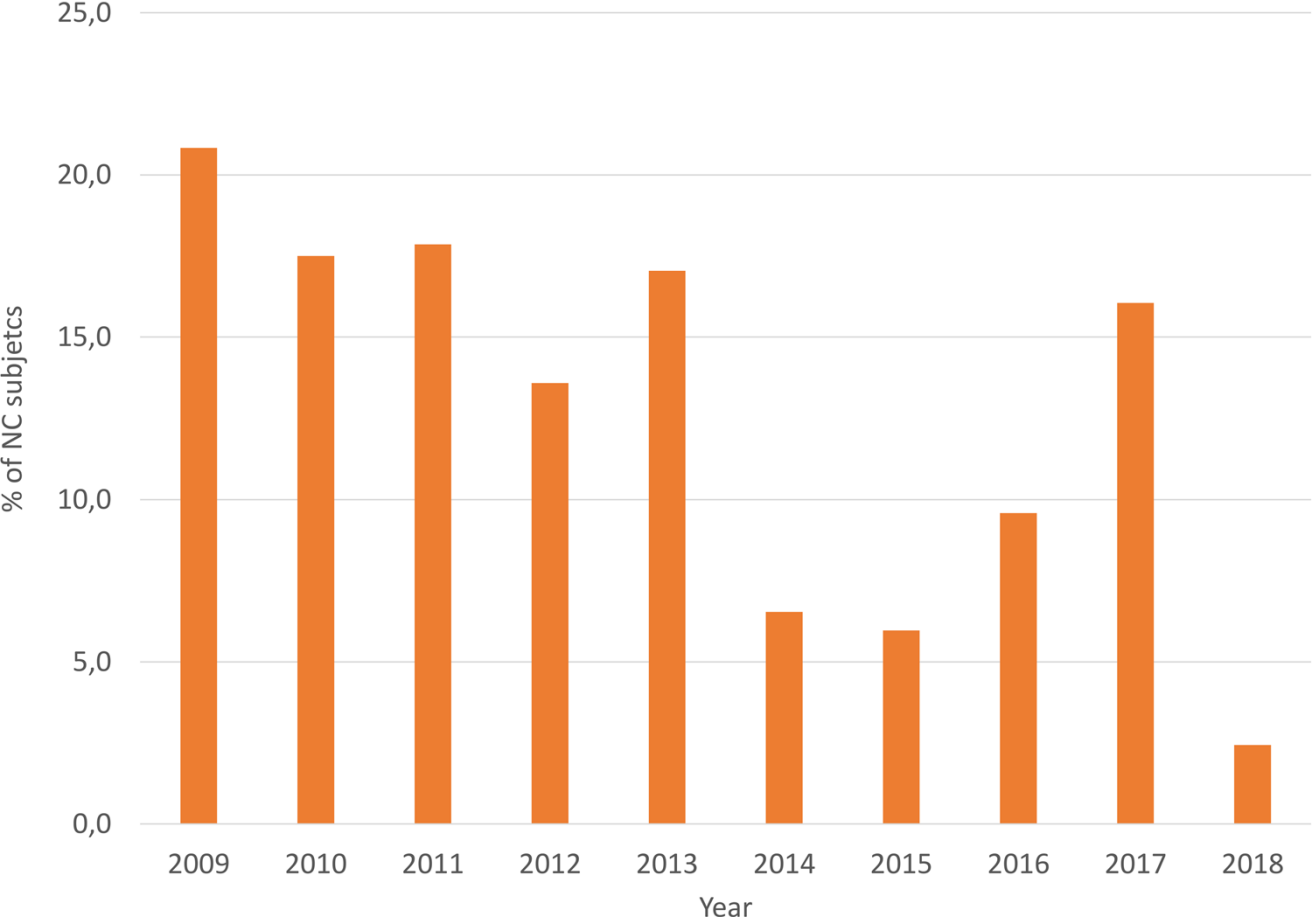
Percentage of carrier (NC) GSPs in Italy by year

In Figure 2, despite the exception of two years (2013 and 2017), the observed trend of the percentage of carriers decreased almost every year. Applying a chi‐square text on the genotypes NN and NC observed in the first five years (from 2009 to 2013) versus the last five years (from 2014 to 2018), we obtained a value highly significant (*p* < 0.001), so that the number of carrier subjects observed from 2014 to 2018 is significantly lower if compared to the one observed in the first five years.

Overall, the mutant allele showed a frequency of almost 6% in the population, with 97 carrier subjects (12.9% of the entire tested cohort). Having available data and pedigree information of the cohort analysed, we investigated if there was any relationship among the mutated allele and the sex of the dogs or their coat colour. A regression logistic analysis has been performed, including sex, coat colour and their interaction, to investigate any possible correlation with the observed genotype and the factors in the model. According to our results, there was no significant interaction between gender and coat colour and no effect of gender of the dogs, while there was a significant effect of coat colour.

## DISCUSSION

In this paper, we showed the genetic trend of a serious genetic pathology, the JEB, in the GSP population in Italy. Considering the total number of GSPs in a span of 10 years in Italy (26,446 registered dogs) and the total number of tests provided (750), a 2.8% of the population has been surveyed. The size of the Italian population of GSPs in the 10 years period showed a negative average growth rate of −3.09%, as shown in Table 1.

According to the genotypes detected, data highlighted a percentage of the C (mutated) allele ≤0.10 on every year. Regarding the genetic trend over time, if we consider that in 2009 there were more than two out of 10 tested subjects with the mutant allele, in 2018 there were about two out of 100 tested subjects with the heterozygous genotype (NC).

The encouraging trend of the reduction of the C allele is confirmed in the first nine months of the 2019, when only one dog out of the 60 tested has been diagnosed as a carrier (data not shown).

The regression logistic analysis, including sex, coat colour and their interaction showed a significant effect of coat colour on the genotypes detected. Dogs with a solid coat colour (whether brown or black) showed more carriers than those with a roan coat (*p*‐value = 0.035). In particular, 16.10% of carrier dogs showed a solid coat colour, while 10.89% had a roan pattern. The percentages are referred to the number of carriers on the total number of solid and roan dogs, respectively (47 NC on 292 solid dog and for 49 NC on 450 roan). It is interesting to notice that the association of the epidermiolysis bullosa and the mottled pigmentation is known^18^ as well as the association of the human gene LAMA3 and the abnormality of skin pigmentation (http://www.genecards.org, Human Phenotype Ontology).

Breeders should carefully observe and declare the coat colour of newborn puppies in order to better comprehend the eventual existence of unusual coat pattern associated with this genotype. It is worth mentioning that 2.8% of the Italian GSPs has been surveyed, and due to the severity of the pathology the genetic test should be performed on every GSP to eradicate the mutant allele from the population, and to confirm the statistical evidence found.

The prevalence of affected GSPs (CC) in Italy is probably underestimated in this study. In the authors’ experience, affected dogs are usually euthanized without being tested because the footpad lesions make them almost unable to walk (as shown in Figure 1), oral lesions render them unable to eat, or because they develop early onset septicaemia, with a poor prognosis.

In conclusion, the simplicity and the low cost of the analysis for the detection of this pathology suggests that a wider population screening and identification of carrier dogs will allow better breeding strategies and management, leading to a rapid JEB eradication. This simple genetic analysis can efficiently increase the welfare of the GSP in Italian population.

The statistical evidence of the higher observation number of NC genotype in solid coat colour dogs must be carefully evaluated in a wider cohort, keeping in mind that the objective of a judicious selection is to keep the genetic pool as wide as possible. Genomic analyses of the population could add useful information to explain the evidence found. The identification of healthy carrier dogs is the only way to enable control of the presence of JEB in the GSP breed by applying an appropriate breeding strategy.

## CONFLICT OF INTEREST

The authors declare no conflict of interest.
